# Eliciting harms data from trial participants: how perceptions of illness and treatment mediate recognition of relevant information to report

**DOI:** 10.1186/1745-6215-12-S1-A10

**Published:** 2011-12-13

**Authors:** Elizabeth N Allen, Karen I Barnes, Adiel Mushi, Isolide Massawe, Sarah G Staedke, Ushma Mehta, Lasse S Vestergaard, Martha M Lemnge, Clare I Chandler

**Affiliations:** 1 Division of Clinical Pharmacology, Department of Medicine, University of Cape Town, Cape Town, South Africa; 2 National Institute of Medical Research, Dar es salaam, Tanzania; 3 National Institute of Medical Research, Tanga Centre, Tanga, Tanzania; 4 Department of Clinical Research, London School of Hygiene and Tropical Medicine, London, UK; 5 Independent Pharmacovigilance Consultant, Johannesburg, South Africa; 6 Centre for Medical Parasitology at the Department of International Health, Immunology and Microbiology, University of Copenhagen, Denmark; 7 Department of Global Health and Development, London School of Hygiene and Tropical Medicine, London, UK

## Background

There is no consensus on the ideal methodology for eliciting participant-reported harms, but question methods influence the extent and nature of data detected. This gives potential for measurement error and undermines meta-analyses of adverse effects. We undertook to identify barriers to accurate and complete reporting of harms data, by qualitatively exploring participants’ experiences of illness and treatment, and reporting behaviours; and compared the number and nature of data detected by three enquiry methods.

## Methods

Participants within antiretroviral/antimalarial interaction trials in South Africa and Tanzania were asked about medical history, treatments and/or adverse events by general enquiries followed by checklists. Those reporting differently between these two question methods were invited to an in-depth interview and focus group discussion. Health narratives were analysed to investigate accuracy and completeness of case record form data and to understand reasons for differential reporting between question methods. Outcomes were the number and nature of data by question method, themes from qualitative analyses and a theoretical interpretation of participants’ experiences.

## Results

We observed a cumulative increase in sensitivity of detection of all types of reports while progressing from general enquiry, through checklist, to in-depth interview. Questioning detail and terminology influenced participants’ recognition of health issues and treatments. Reporting patterns and vocabulary suggest influence from the relative importance that illnesses and treatments have for participants. Perceptions were often dichotomised (e.g. ‘street’ versus clinic treatments, symptoms experienced versus tests and examinations performed, chronic versus acute illness, persistent versus intermittent symptoms, activity- versus malaria-related symptoms) and this differentiation extended to ideas of relevance to report. South African participants displayed a ‘trial citizenship’, taking responsibility for the impact of their reporting on trial results, and even reaching reporting decisions by consensus. In contrast, Tanzanians perceived their role more as patients than participants; the locus of responsibility for knowing information relevant to the trial fell with trial staff as doctors rather than with themselves.

## Conclusions

Our observations of how reporting relates to participant perceptions inside and outside trials could help optimise how harms data are elicited. Questions reflecting the different ways that biomedically defined illness and treatment data are perceived by participants may help them understand relevance for reporting. We will theorise how these two disparate trial environments may have influenced how participants understood their role, as this could help researchers achieve empowered participation in similar trials.

